# Supplementary material for: Parity-dependent state transfer for direct entanglement generation
Source: Nat Commun. 2025 Mar 18;16:2660. doi: 10.1038/s41467-025-57818-2 (PMC11920060; doi:10.1038/s41467-025-57818-2)
Supplement: Supplementary file 1 — Supplementary Information [file 41467_2025_57818_MOESM1_ESM.pdf]

Parity-dependent state transfer for direct entanglement generation – Supplementary Information –

Supplementary Note 1 – Experimental setup

A schematic of the experimental setup is shown in Supplementary Fig. 1**a**. The qubit MW control pulses are generated using a Zurich Instruments (ZI) “Super High Frequency Quantum Analyzer” (SHFSG). The MW drive lines are attenuated by −60 dB distributed over the different temperature stages and routed to the individual qubit drive lines on the device. Flux biasing and parametric drives are applied simultaneously using a ZI “High Density Arbitrary Waveform Generator” (HDAWG). The flux lines are attenuated by −20 dB, filtered using a 780 MHz or 650 MHz low-pass filter (Mini-Circuits VLFX-780+ or VLFX-650+) and routed to the individual coupler flux lines on the device. The measurement pulses are generated using a ZI “Super High Frequency Quantum Analyzer” (SHFQA), allowing us to multiplex different readout tones on the same signal. The measurement signal lines are attenuated by −70 dB distributed over the different temperature stages, filtered using a 5 GHz high-pass filter (Mini-Circuits VHF5050+) and routed to the two feedlines on the device. The output signals from the feedlines are filtered using a 5 GHz high-pass filter, routed through four 4–12 GHz isolators and 0 dB attenuators for thermal connection at each stage, then amplified by a 4–8 GHz 40 dB HEMT cryogenic low-noise amplifier (LNF-LNC4_8F) on the 4 K stage and a 40 dB low-noise room temperature amplifier (BZ-04000800-081045-152020), before being routed back to the SHFQA for analysis. The chip is housed in a 16-port package with a copper cavity. Signals are launched to a multi-layer Isola Astra PCB with a 16-port Ardent connector, shown in Supplementary Fig. 1**b**-**c**, and routed from the PCB traces to the chip with wire bonds. The properties of qubits, resonators and couplers are given in Table 1. Qubit coherence times are measured with all tunable couplers positioned at their respective upper sweet spots. Notably, qubits q_2, q_5 and q_6 are affected by charge dispersion due to lower E_J/E_C ratios ($\lesssim 36$), which lead to beating patterns in Ramsey experiments. From these, we extract the decay rate Γ and the charge dispersion, resulting in beating frequencies Δ in the range of 70-300 kHz, and determine the effective T_2^* as the time when the envelope $e^{-\Gamma t}\cos(\Delta t)$ first reaches the $1/e$ threshold.

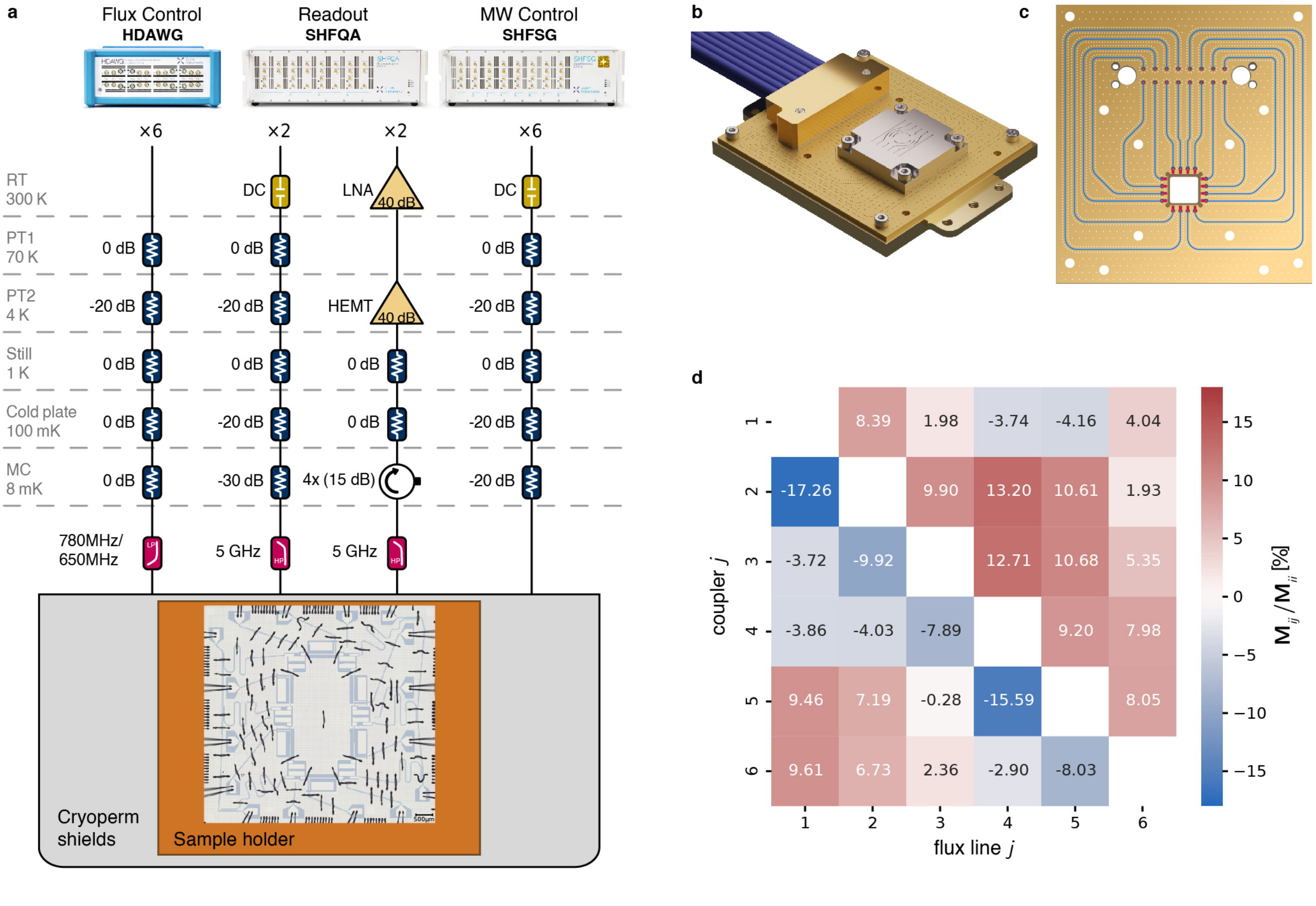

Supplementary Fig. 1 | Experimental setup and DC flux crosstalk. a Wiring and control electronics of the experiment. Schematic of the qubit housing showing the PCB with Ardent connector and copper sample holder **b** and the routing of the PCB **c**. See main text for details. **d** Flux crosstalk matrix: entries correspond to the ratio $\mathbf{M}_{ij}/\mathbf{M}_{ii}$, given in percentage, where $\mathbf{M}_{ij}$ is the required change in voltage applied to line j in order to induce a change of one flux quantum in the flux threading the SQUID loop of coupler i.

i	1	2	3	4	5	6
Qubit frequency, ω_q	4.37 GHz	3.93 GHz	4.27 GHz	4.23 GHz	3.83 GHz	3.21 GHz
Qubit anharmonicity, α_q	−247.3 MHz	−249.4 MHz	−249.7 MHz	−252.1 MHz	−250.6 MHz	−255.1 MHz
Resonator frequency, ω_r	6.04 GHz	5.97 GHz	6.04 GHz	6.11 GHz	6.15 GHz	6.17 GHz
Coupler frequency range, ω_c	3.65-7.17 GHz	4.92-7.51 GHz	3.38-7.28 GHz	4.66-6.75 GHz	2.57-4.71 GHz	3.95-6.93 GHz
Relaxation time, T_1	12.1 ± 2.4 µs	53.2 ± 7.3 µs	26.2 ± 3.4 µs	46.0 ± 8.6 µs	63.4 ± 9.4 µs	72.0 ± 7.0 µs
Ramsey decay time, T_2^*	10.0 ± 2.0 µs	8.1 ± 6.5 µs	8.1 ± 1.1 µs	7.0 ± 1.0 µs	4.3 ± 1.7 µs	4.1 ± 1.2 µs
Readout fidelity F_{RO}	78.3 ± 0.6 %	91.3 ± 0.1 %	89.4 ± 1.6 %	87.4 ± 0.9 %	81.8 ± 1.1 %	80.0 ± 0.9 %
single-qubit RB error ϵ_{1q}	0.20 ± 0.02 %	0.23 ± 0.01 %	0.11 ± 0.02 %	0.32 ± 0.06 %	0.20 ± 0.01 %	0.053 ± 0.003 %
ZZ coupling ζ_i	-108 ± 27 kHz	-362 ± 8 kHz	816 ± 14 kHz	-273 ± 8 kHz	-18 ± 27 kHz	-31 ± 26 kHz
Qubit - Next Coupler, $g_{i,j=i}$	62 MHz	74 MHz	68 MHz	60 MHz	47 MHz	77 MHz
Qubit - Prev. Coupler, $g_{i,j=i-1}$	65 MHz	59 MHz	112 MHz	65 MHz	61 MHz	64 MHz
Qubit - Qubit, $g_{i,i+1}$	–	6.0 MHz	8.3 MHz	6.6 MHz	4.8 MHz	–

Supplementary Table 1 | System parameters.

The system exhibits large flux crosstalk due to undesired inductive coupling of SQUID loops to distant flux lines. We characterise the mutual inductance between all flux lines and couplers by performing spectroscopy measurements on the resonators and qubits, while varying the DC voltage bias on each line [1–3]. The flux experienced by each coupler is given by

$$\bar{\Phi} = \mathbf{M}\bar{V} + \bar{\Phi}_{\text{off}} \tag{1}$$

where $\bar{V}_j$ is the voltage bias applied to flux line j and $\bar{\Phi}_i$ is the flux experienced by the coupler i. The matrix element M_{ij} characterises the change in voltage required on line j to vary the flux experienced by coupler i by one flux quantum. The vector $\bar{\Phi}_{\text{off}}$ accounts for additional flux sources present in the environment. For a desired flux operation point of the couplers, the required DC bias $\bar{V}$ is calculated by simply inverting Eq. (1). The flux crosstalk in the device is determined by normalising the values M_{ij} by M_{ii}, as shown in Supplementary Fig. 1**d**.

Supplementary Note 2 – PST in chains with different length

We perform PST operations on chains of varying length N by applying the calibration method described in the main text to different qubit subsets. For each calibrated transfer, dynamics were probed for all possible initial locations by applying the simultaneous drive for varying duration and measuring all qubit populations. Dynamics for exemplary chains composed of three through six qubits are shown in Supplementary Fig. 6. Equivalently to the subset of plots showed in the main text, these are compared to simulation of the PST Hamiltonian with ideal coupling strengths and including decay rates as non-Hermitian terms. Calibrated transfer times τ_N for the chains here depicted are $\tau_3 = 216$ ns, $\tau_4 = 429$ ns, $\tau_5 = 500$ ns and $\tau_6 = 640$ ns.

Supplementary Note 3 – Circuit for GHZ creation

As discussed in the main text, PST enables the direct generation of GHZ states. This is achieved by preparing all qubits in the superposition state, applying PST to realise multiple simultaneous transfers with parity-dependent phases and mapping the resulting state onto the GHZ state via a final layer of single-qubit gates. We derive the exact gates required in the final layer by using a sequence of local transformations to well-known graph states [4]. Firstly, we map the state generated after the PST operation (Supplementary Fig. 2**a**) to the all-to-all connected graph state (Supplementary Fig. 2**b**) by applying a $Z_{-\pi/2}$ on all qubits except the center qubit in a odd chain, which accounts for

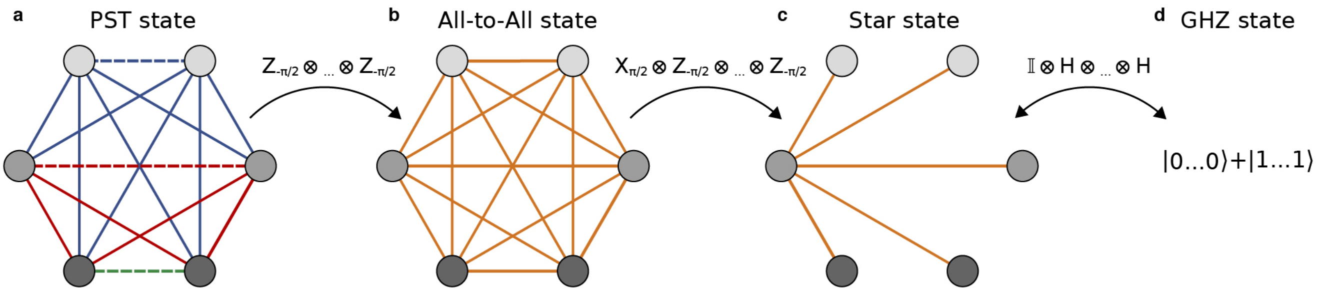

Supplementary Fig. 2 | Steps of derivation for GHZ circuit with six qubits. By using only single-qubit operations, the entangled state obtained by applying the PST operation to an equal superposition state **a** can be mapped to well-known graph states **b-c** and ultimately to the GHZ state **d**. Labels above each black arrow show the required local unitary for each step.

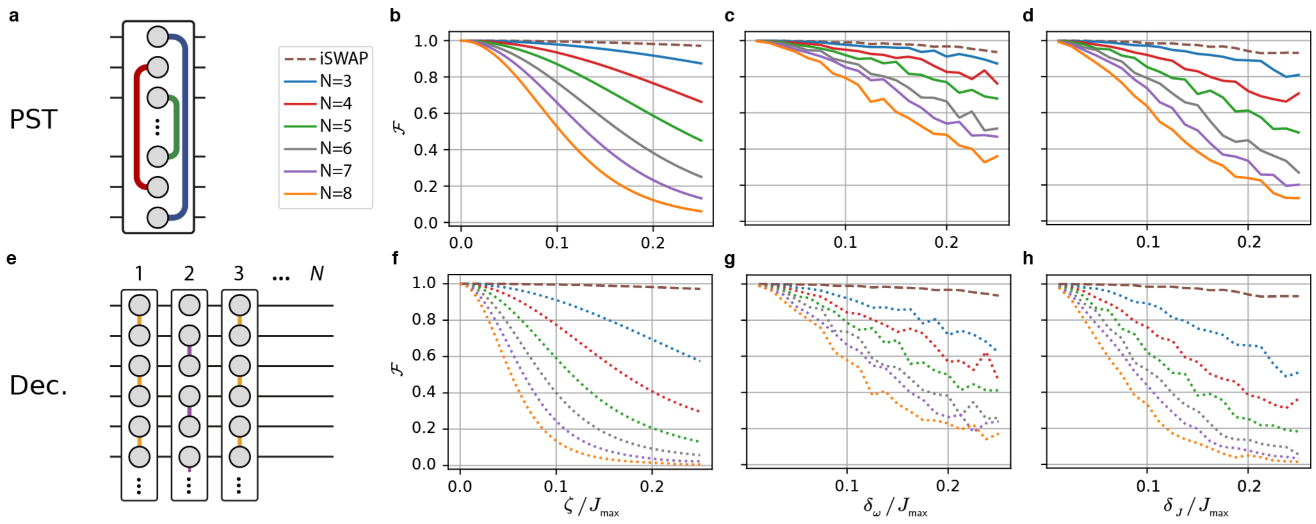

Supplementary Fig. 3 | Error sensitivity of the PST operation and its equivalent decomposition. Unitary fidelity overlap with ideal PST operation as a function of ZZ-type errors **b**,**f**, detuning errors **c**,**g** and coupling errors **d**,**h**. The top row shows the errors for PST (shown in **a**) and the bottom row for its equivalent decomposition into the repeated application of two-qubit iSWAP gates (shown in **e**). ZZ errors are applied with equal strength on the whole chain. Detuning and coupling errors are sampled from normal distributions with standard deviation δ_ω^i and δ_J^i, respectively. 100 realizations of the noise are averaged in the numerical simulation for each data point.

the iSWAP-like operation between mirror-symmetric qubits. Using local equivalence of graph states, we then obtain the star graph state (Supplementary Fig. 2**c**), composed of a central node connected to all others. Here, a $Z_{-\pi/2}$ gate is applied on all qubits, except the qubit chosen to be the central node, where a $X_{\pi/2}$ gate is applied instead. Finally, applying a Hadamard gate on all qubits except the central node transforms the star graph state into the GHZ state (Supplementary Fig. 2**d**). Collectively, these transformations result in a final layer of single-qubit gates comprising $Y_{\pi/2}$ rotations on all qubits, except for: an arbitrary qubit in even chains, where instead a $Z_{-\pi/2}$ followed by an $X_{\pi/2}$ are applied; the centre qubit in odd chains, where instead a $X_{\pi/2}$ rotation is applied (choosing the central node of the star state to coincide with the centre qubit).

Supplementary Note 4 – Robustness of PST and optimal decomposition

The decomposition of the PST into two-qubit gates operation discussed in the main text provides an intuitive understanding and a clear mapping to the graph state representation. However, a more efficient decomposition exists based on the alternating application of iSWAP gates [5], shown in Supplementary Fig. 3**e**. Derived in the context of Fermi-Hubbard simulations [6, 7], this gate sequence is, to the best of our knowledge, the most time- and gate-efficient decomposition of the PST operation. Nonetheless, PST is at least a factor of two faster than this efficient decomposition, as $\tau_{\text{PST}} \leq \frac{N\pi}{4J_{\max}}$ for a chain length N, and the decomposition takes $\tau_{\text{decomp}} = N \times \tau_{\text{iSWAP}} = \frac{N\pi}{2J_{\max}}$. The increased speed of PST improves its robustness to relaxation and dephasing errors, since these scale linearly with time in the small error limit. On the other hand, the effect of coherent errors on both implementations is non-trivial. Therefore we perform simulations in the presence of additional error terms in the chain Hamiltonian

$$\hat{H}_{\text{err}}/\hbar = \sum_{i=1}^{N-1} \frac{\zeta}{4}(\mathbb{1}_i\mathbb{1}_{i+1} - \mathbb{1}_i\sigma_{i+1}^z - \sigma_i^z\mathbb{1}_{i+1} + \sigma_i^z\sigma_{i+1}^z) + \sum_{i=1}^{N} \Delta_\omega^i \sigma_i^z + \sum_{i=1}^{N-1} \Delta_J^i(\hat{\sigma}_i^-\hat{\sigma}_{i+1}^+ + \hat{\sigma}_i^+\hat{\sigma}_{i+1}^-), \tag{2}$$

where the errors $\Delta_\omega^i, \Delta_J^i$ are randomly sampled from normal distributions with standard deviations $\delta_\omega^i, \delta_J^i$. We then calculate the fidelity of each implementation from the unitary overlap with the ideal operation $\mathcal{F} = |\text{Tr}(U_{\text{err}}U_{\text{ideal}}^\dagger)/d|^2$, where $d = 2^N$ is the dimension of the Hilbert space. Unwanted ZZ interactions produce phase errors accumulating over time and, therefore, affect the PST operation less than its decomposition, as shown in Supplementary Fig. 3**b** and **f**. PST also shows increased robustness to detuning and coupling errors, as shown in Supplementary Fig. 3**c-d** and **g-h**. These results demonstrate that the PST operation is more robust than its decomposition to a variety of error sources in spite of the added complexity of the dynamics.

While the sequence discussed here is the exact decomposition of the PST operation, the gate-optimal sequence for the generation of GHZ states is a single Hadamard gate followed by CNOT gates to entangle each following qubit. To minimise the total duration, the sequence can be started in the middle of the chain, and then, after the first CNOT, at each step, two CNOT gates can be applied simultaneously, one on each side. The total operation time is

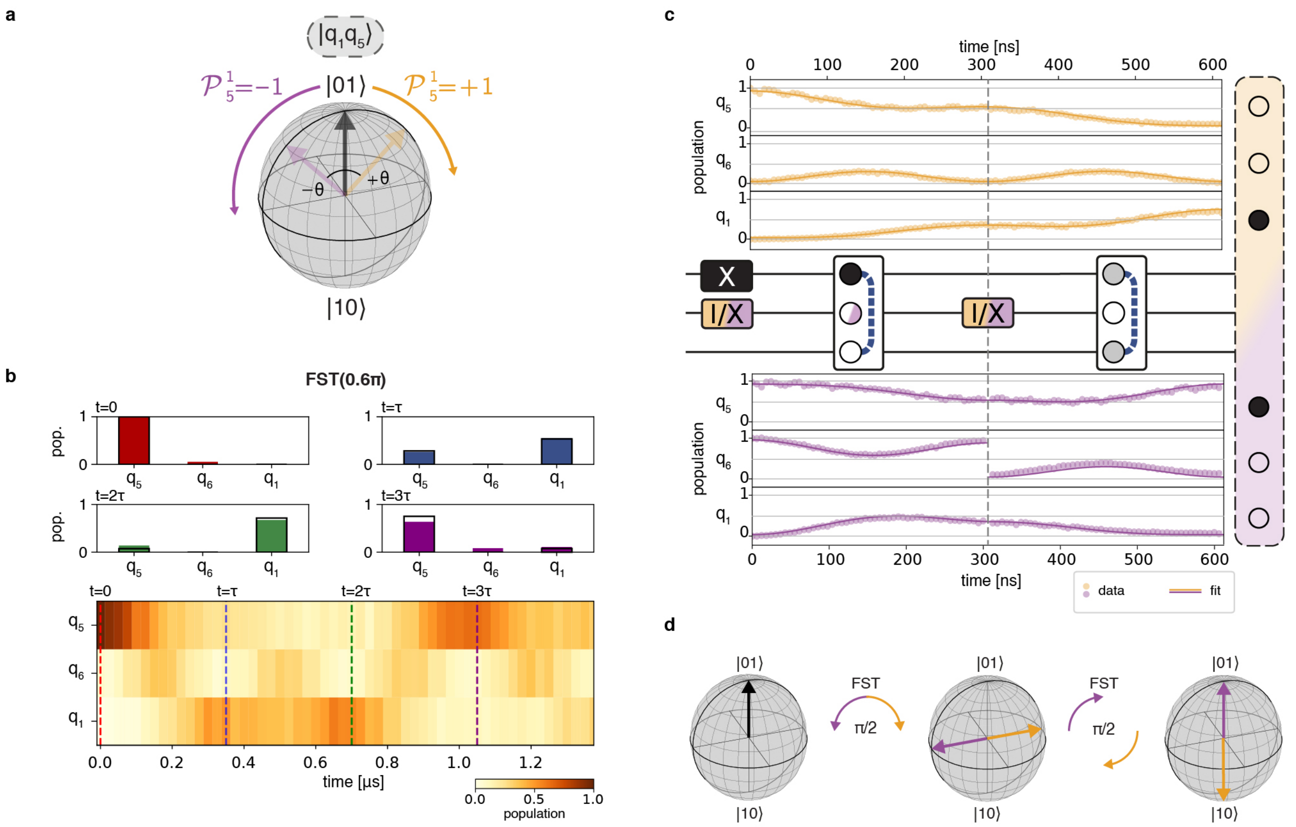

Supplementary Fig. 4 | Fractional state transfers on a three-qubit chain. a Bloch sphere visualisation of FST. The fractional transfer results in a rotation in the Bloch sphere spanned by the single-excitation states of the two outer qubits. The rotation angle $\pm\theta$ depends on the presence (orange) or absence (purple) of an excitation in the center qubit. **b** Single-excitation dynamics of FST for $\theta = 0.6\pi$. The excitation is partially transferred between the two outer qubits after integer multiples of the transfer time $\tau = 350\,\text{ns}$. Contour plot (bottom) shows the dynamics, and solid-bar plots highlight the excited state populations of all qubits for $t = 0, \tau, 2\tau, 3\tau$ (top). Black wireframes show the expected distribution renormalised by the experimentally observed decay. **c** Parity-dependence on double-transfer dynamics. Qubits populations when preparing a single excitation on one of the outer qubits and applying FST($\pi/2$) twice. Measurement are repeated for the center qubit prepared in the ground state (orange plot and gates) or the excited state (purple plot and gates) during the first FST($\pi/2$). Dots represent measured population and solid lines show simulated dynamics under the fitted chain Hamiltonian from Eq. (3). **d** Bloch sphere visualisation of the double-transfer experiments. State of qubits q_1 and q_5 are represented on Bloch sphere for different instances in the sequence: after state preparation (left), after the first FST($\pi/2$) operation (centre) and after the second one (right). Colors represent the parity during the first transfer.

then given by $\lceil N/2 \rceil \times \tau_{\text{CNOT}}$. Assuming that the CNOT operation is implemented using a CZ gate and Hadamards, $\tau_{\text{CNOT}} > \sqrt{2}\tau_{\text{iSWAP}}$. Then, as this sequence takes at least $\frac{N}{2} \times \tau_{\text{CNOT}} > \frac{N}{\sqrt{2}} \times \tau_{\text{iSWAP}}$, PST still provides a speed improvement of at least a factor of $\sqrt{2}$. Hence, we expect similar fidelity improvements as described above.

Supplementary Note 5 – Fractional State Transfer

Fractional State Transfer (FST) can be viewed as a generalised form of PST, granting partial transfer between mirror-symmetric qubits by an arbitrary amount. In our system, FST is achieved by allowing the parametric drive frequencies (ω_{d_n}) to be detuned from the difference frequency of the respective adjacent qubits (Δ_n). Revisiting the procedure described in the main text yields the chain Hamiltonian

$$\hat{H}_{\text{chain}}/\hbar = -\sum_{n=1}^{N} \frac{\delta_n}{2}\hat{\sigma}_n^z + \sum_{n=1}^{N-1} J_n(\hat{\sigma}_n^- \hat{\sigma}_{n+1}^+ + \text{h.c.}), \tag{3}$$

where (δ_n) are the qubit frequencies in the drive's frame, satisfying $\delta_{n+1} - \delta_n = \Delta_n - \omega_{\text{d}_n}$. A FST for an arbitrary transfer angle θ is obtained by setting the coupling strengths and qubit frequencies as

$$\delta_n = \begin{cases} 0 \\ \frac{\pi}{2\tau}\left(\frac{\theta}{\pi} - 1\right)\frac{N}{2}\left(\frac{1}{2n-N} - \frac{1}{2n-2-N}\right) \end{cases} \qquad J_n = \begin{cases} \frac{\pi}{2\tau}\sqrt{\frac{n(N-n)((N-2n)^2-(\frac{\theta}{\pi})^2)}{(N-1-2n)(N+1-2n)}} & \text{for } N \text{ even,} \\ \frac{\pi}{2\tau}\sqrt{\frac{n(N-n)((N-2n)^2-(\frac{\theta}{\pi}-1)^2)}{(N-2n)^2}} & \text{for } N \text{ odd,} \end{cases} \tag{4}$$

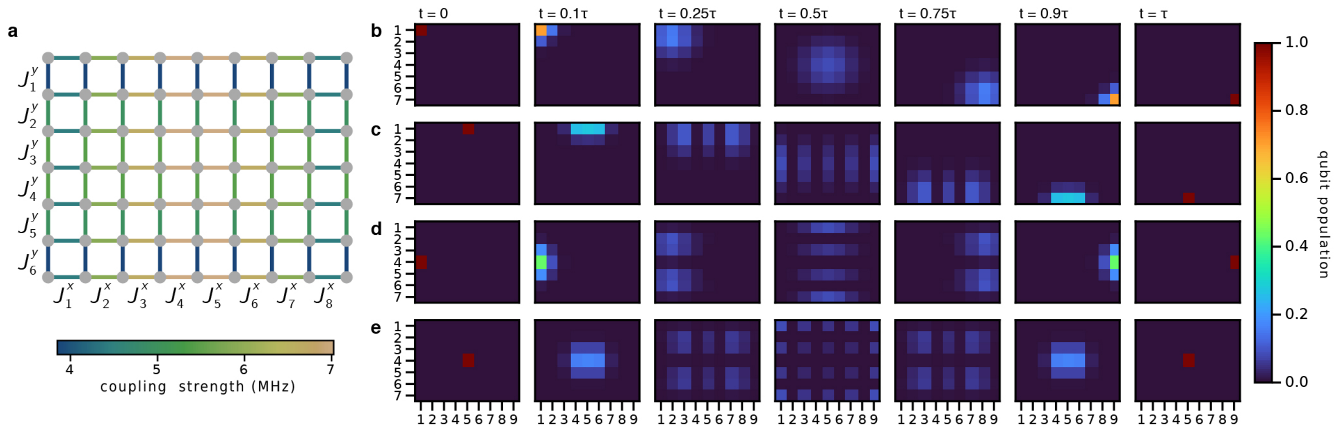

Supplementary Fig. 5 | Simulations of PST in a two-dimensional square lattice. a Two-dimensional 9-by-7 qubit square lattice with couplings engineered for PST with a transfer time of $\tau = 1\,\mu\text{s}$. Couplings strengths from position $(x = i, y)$ to $(x = i+1, y)$ are given by J_i^x and from position $(x, y = j)$ to $(x, y = j+1)$ are given by J_j^y. **b-e** Simulated dynamics of excitations started at different positions of the lattice, shown at times $t = 0, 0.1\tau, 0.25\tau, 0.5\tau, 0.75\tau, 0.9\tau$ and τ.

resulting in the coherent transfer of the fraction $\sin^2(\theta/2)$ of an excitation between any qubit and its mirror-symmetric counterpart. Equivalently to PST, the FST protocol can also be described by an effective Hamiltonian [5]

$$\hat{H}_{\mathrm{FST}}/\hbar = \frac{\theta}{2\tau} \sum_{n=1}^{\lfloor \frac{N}{2} \rfloor} \left(\bigotimes_{k=n+1}^{\tilde{n}-1} \hat{\sigma}_k^z \right) (\hat{\sigma}_n^- \hat{\sigma}_{\tilde{n}}^+ + \mathrm{h.c.}). \tag{5}$$

whose dynamics are stroboscopically equivalent to the dynamics described by Eq. (3). For every mirror-symmetric pair of qubits q_n and $q_{\tilde{n}}$, the unitary evolution $U = e^{-i\hat{H}_{\mathrm{FST}}\tau/\hbar}$ describes a rotation by a transfer angle of $\pm\theta$ in the Bloch sphere spanned by the two single-excitation states, as shown in Supplementary Fig. 4**a**. The sign of the rotation angle depends on the number parity of excitations between q_n and $q_{\tilde{n}}$, once again described by the operator $\hat{\mathcal{P}}_{n+1}^{\tilde{n}-1} = \bigotimes_{k=n+1}^{\tilde{n}-1} \hat{\sigma}_k^z \in \{-1, 1\}$.

We implement FST on a chain of $N = 3$ qubits, q_5, q_6 and q_1, with a transfer time $\tau = 350\,\text{ns}$ and transfer angle $\theta = 0.6\pi$, as shown in the dynamics of Supplementary Fig. 4**b**. Solid-bar plots highlight the population on each qubit for multiples of the transfer time τ. The excitation only partially transfers between the outer qubits and matches the expected distributions, shown as black wireframes.

In order to highlight the parity-dependence property of FST, we perform two experiments involving repeat applications of an FST with transfer angle $\theta = \pi/2$, shown in Supplementary Fig. 4**c-d**. In both cases, we prepare a full excitation on one of the outer qubits, q_5, followed by two identical FST$(\pi/2)$ operations and a measurement to all qubits. In the second experiment, however, an extra excitation is initially prepared on the middle qubit q_5 and removed right before the second FST$(\pi/2)$ operation by an additional X_π pulse. Because $\langle \hat{\mathcal{P}}_5^1 \rangle = 1$ for both FST operations in the first sequence, the corresponding Bloch sphere rotations happen in the same direction and amount to a full population transfer to the mirror-symmetric qubit q_1. In the second sequence, however, the two transfers produce rotations with opposite directions since $\hat{\mathcal{P}}_5^1$ changes from negative to positive, resulting in the excitation being refocused back to the original qubit, q_5.

Supplementary Note 6 – PST on square lattices

The simplest extension of qubit chains to higher dimensions is square lattices with arbitrary dimensions. To achieve PST in these structures, the couplings are engineered so as to satisfy the PST formula $J_n = \frac{\pi}{2\tau}\sqrt{n(N-n)}$ for every axis direction with equivalent transfer times τ [8], as shown in Supplementary Fig. 5**a** for a two-dimensional square lattice. Here, J_i^x and J_j^y represent the coupling strengths between positions from column i to column $i+1$ and from row j to row $j+1$, respectively. Due to this structure the two dimensions behave independently, with PST happening simultaneously on each dimension, as shown in simulation for different initial excitation locations in Supplementary Fig. 5**b-e**. Note that, since the Jordan-Wigner transformation does not apply in higher dimensions, here PST is no longer ensured when multiple excitations are present in the lattice.

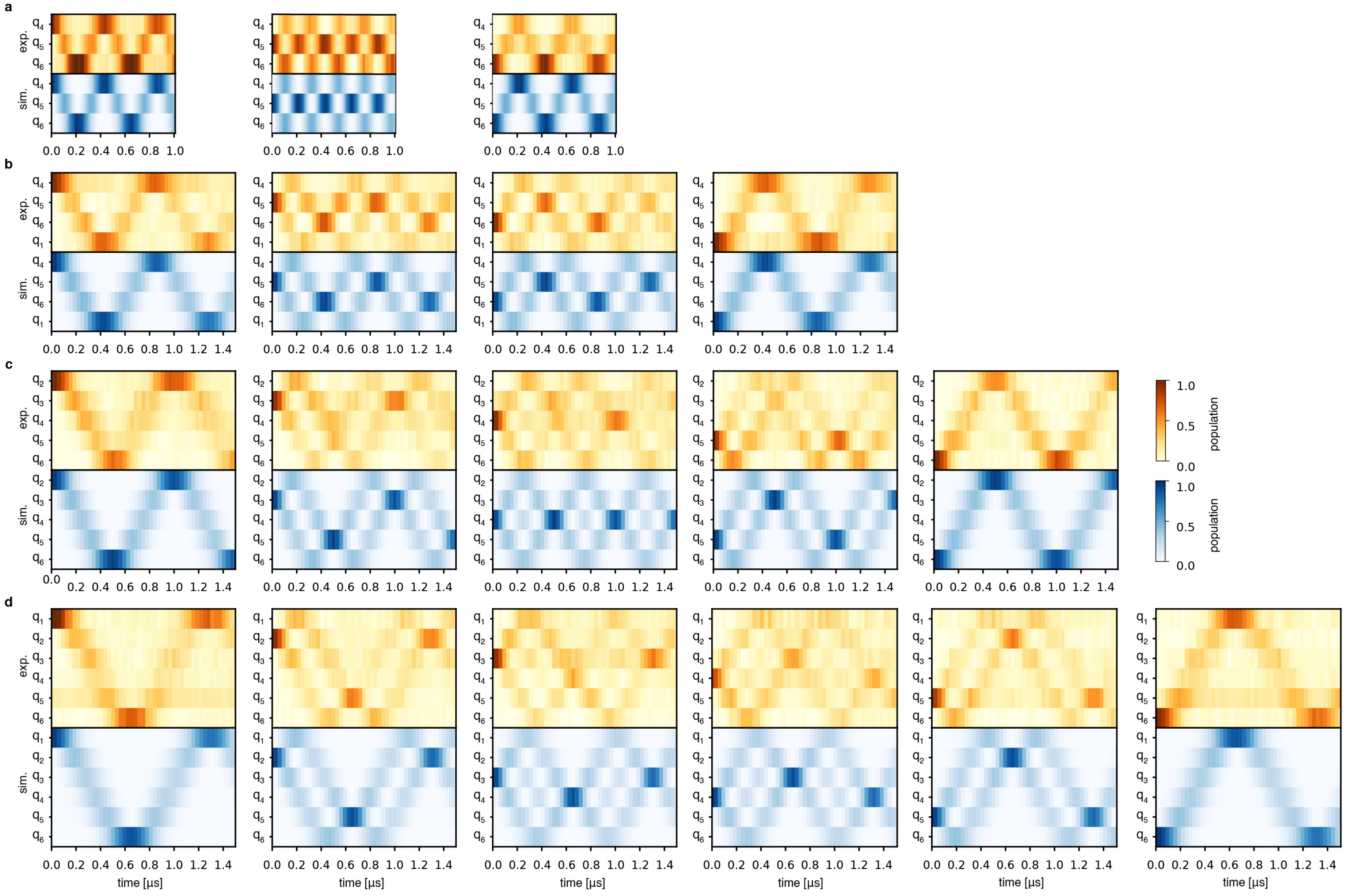

Supplementary Fig. 6 | Perfect State Transfer protocol for various chain lengths. All single-excitation dynamics are shown for illustrative cases with three **a**, four **b**, five **c** and six **d** qubits. For each chain, experimental data (orange) is compared to simulation with ideal coupling strengths and relaxation effects for the respective qubit subset (blue).

Supplementary References

[1] D. M. Abrams, N. Didier, S. A. Caldwell, B. R. Johnson, and C. A. Ryan, *Methods for Measuring Magnetic Flux Crosstalk between Tunable Transmons*, Phys. Rev. Appl. **12**, 064022 (2019).

[2] X. Dai, D. Tennant, R. Trappen, A. Martinez, D. Melanson, M. Yurtalan, Y. Tang, S. Novikov, J. Grover, S. Disseler, et al., *Calibration of Flux Crosstalk in Large-Scale Flux-Tunable Superconducting Quantum Circuits*, PRX Quantum **2**, 040313 (2021).

[3] C. N. Barrett, A. H. Karamlou, S. E. Muschinske, I. T. Rosen, J. Braumüller, R. Das, D. K. Kim, B. M. Niedzielski, M. Schuldt, K. Serniak, et al., *Learning-Based Calibration of Flux Crosstalk in Transmon Qubit Arrays*, Phys. Rev. Appl. **20**, 024070 (2023).

[4] M. Hein, J. Eisert, and H. J. Briegel, *Multiparty entanglement in graph states*, Phys. Rev. A **69**, 062311 (2004).

[5] M. Nägele, C. Schweizer, F. Roy, and S. Filipp, *Effective nonlocal parity-dependent couplings in qubit chains*, Phys. Rev. Res. **4**, 033166 (2022).

[6] I. D. Kivlichan, J. McClean, N. Wiebe, C. Gidney, A. Aspuru-Guzik, G. K.-L. Chan, and R. Babbush, *Quantum Simulation of Electronic Structure with Linear Depth and Connectivity*, Phys. Rev. Lett. **120**, 110501 (2018).

[7] C. Cade, L. Mineh, A. Montanaro, and S. Stanisic, *Strategies for solving the Fermi-Hubbard model on near-term quantum computers*, Phys. Rev. B **102**, 235122 (2020).

[8] A. Kay, *Perfect, efficient, state transfer and its application as a constructive tool*, International Journal of Quantum Information **8**, 641 (2010).
