# Supplementary material for: Parity-dependent state transfer for direct entanglement generation
Source: Nat Commun. 2025 Mar 18;16:2660. doi: 10.1038/s41467-025-57818-2 (PMC11920060; doi:10.1038/s41467-025-57818-2)
Supplement: Supplementary file 2 — Transparent Peer Review file [file 41467_2025_57818_MOESM2_ESM.pdf]

nature portfolio

Peer Review File

Parity-dependent state transfer for direct entanglement generation

Corresponding Author: Mr Federico Roy

This file contains all reviewer reports in order by version, followed by all author rebuttals in order by version.

Version 0:

Reviewer comments:

Reviewer #1

(Remarks to the Author)
In this paper, the author provides an experimental demonstration of Perfect State Transfer (PST) in a 1D chain of qubits containing both zero and non-zero number of excitations. For the case where excitations are present in the system, the authors describe how the final transferred state will include a phase that depends on the parity of the total number of excitations in the chain. The authors then use the parity dependence of PST to generate a 3-qubits GHZ state with 88% fidelity (after accounting for single-qubit Z rotation).

This manuscript provides a nice experimental demonstration of Perfect State Transfer on a superconducting qubit platform, and discusses how one could apply this protocol in the presence of qubit excitations in the chain. The experimental data is of quality and supports the claims by the authors. Overall, I would support the publication of this manuscript in Nature Communications.

Nevertheless I think the following questions could be interesting to address:

1. One advantage of performing this state transfer in the presence of qubit excitations is that it can be done while a state we wish to preserve is present on that chain (otherwise one could just reset the chain and do away with the parity dependence of the state transfer). The fact that one only needs to know about the parity of the qubits in the chain is interesting because in many error correction codes, the parities of the qubits is a known/measured quantity. In this context,
a. Could this state transfer be done through a stabilized repetition code state without learning/disturbing the logical state?
b. What about a stabilized surface code? (some elements of this is addressed in Supplementary Note 5, but perhaps expanding on this protocol specifically in the presence of a stabilized surface code state would be interesting)
c. What is the effect of the state transfer on the qubits in the chain/lattice?
2. In Fig 2, it looks like the closer the qubit is to the center of the chain, the worse the state transfer fidelity is (as judged by the green bar plots in Fig 2 a,b,c). Presumably this has to do with the increased number of “paths” the information is traveling through (1 in a, 2 in b, and 3 in c). Is this expected, and if so how could it be mitigated?
3. In the paper, the use of PST for GHZ state preparation is compared with using “its decomposition into single- and two-qubit gates”. The authors find that PST is twice as fast. Besides this speed comparison, it could be interesting to know how the fidelity of the GHZ state from the two methods is expected to compare. Additionally, it’s worth noting that,if scalability and speed are the main motivating factors, there exist Measurement Based circuits for generating GHZ state which are of constant depth and could merit a mention (since for PST with a given maximum coupling strength, tau is inversely proportional to the chain length)

Reviewer #2

(Remarks to the Author)
Key results

The authors present an experimental demonstration of Perfect State Transfer (PST) in a linear chain of six qubits with various configurations, including PST between all pairs of mirror-symmetric qubits. They also verify the parity dependence of PST with multiple excitations in the chain. Building on this parity dependence, the authors introduce a novel method for

efficiently generating multi-qubit GHZ states in a system with only nearest-neighbor coupling. As a proof of concept, a three-qubit GHZ state is generated with a state fidelity of 88%.

Significances

This experiment showcases PST with some configurations that are not explored before, including the case where initial population at mid of the chain or transferring with multiple excitation within the chain. The application of PST for GHZ state generation could garner significant attention within the superconducting quantum computing community, as it offers a novel and efficient approach to non-local operations in superconducting circuits. However, it is important to note that PST is a well-established technique that has been extensively studied, both theoretically and experimentally. In fact, a similar, more challenging experiment has recently been published in Nature Communications, see the reference 51 in the main text. While the results here are interesting, they do not represent a significant advancement over prior work.

Presentation, Clarity and Validity

The manuscript is clearly written, and the experimental methods are presented in a comprehensible manner. The primary conclusions are well-supported by experimental data, which are collected and presented in a valid way. However, several key technical details are missing from the manuscript, as outlined in the comments to the authors below.

Comments to authors

1. While the manuscript successfully demonstrates parity-dependent PST across six qubits, the GHZ state generation is limited to three qubits. It would be helpful to include a discussion of the challenges in scaling this protocol to more qubits, as scalability is an important factor in evaluating the protocol's potential for practical use.
2. The authors attribute a significant portion of the error to residual ZZ interactions among qubits. However, a quantitative description of the strength of these residual ZZ interactions is lacking.
3. Many of the results are presented in terms of qubit populations or quantum state tomography. However, the manuscript does not specify the readout fidelity, whether assignment errors were corrected before reconstructing the density matrix, or whether the density matrix reconstruction was done under physical constraints.
4. Additionally, the quality of single-qubit operations should be discussed, as it is relevant for validating the quantum state tomography.
5. Could the authors clarify the meaning of the vertical bars in Fig. 3c?
6. The effective Hamiltonian assumes the qubits as ideal two-level systems, but transmon qubits have only weak anharmonicity. At what point should higher energy levels be considered, and do they impose any limitations on the speed of PST operations?
7. Figure 4a-d presents a novel illustration of how PST induces all-to-all connectivity and generates a GHZ state. However, this section is somewhat unclear. According to the figure, generating a six-qubit GHZ state requires two PST operations and an additional iSWAP operation between qubits 3 and 4. This seems to contradict a statement on line 288 of the main text, which claims "a single PST operation can be utilised to directly generate a GHZ state for any chain length."
8. According to Supplementary Table 1, the qubits used in the experiment have fixed frequencies, yet they exhibit substantial dephasing, with the shortest Ramsey decay time being only 6 µs. How does this dephasing affect the PST process?
9. The lowest qubit frequency in the experiment is 3.21 GHz, making it more susceptible to thermal noise. The manuscript would benefit from a discussion of how thermal noise may have impacted the experimental results.

Reviewer #3

(Remarks to the Author)
This is a nice paper demonstrating perfect state transfer in a superconducting system utilizing fixed-frequency transmons with tunable couplers. A highlight of the work is the entanglement generation via parity-dependent state transport. While the authors only generated a three-qubit GHZ state, I like the experimental protocol because it provides a novel alternative to quantum circuits using two-qubit gates. The paper is well written and the data are convincing, however I would like to see more clarifications on the following points.

1. The qubit-qubit coupling are engineered by pulsing on the tunable coupler with AC and DC components described in line 93. How is the DC component chosen? The AC component is modulated at the k-th harmonic, what k is used experimentally for each coupling? Supp. Fig. 1 shows a 650/750 MHz filter on the flux line which is more than enough to drive the k=1 harmonics for all pairs of qubits, is there any reason to not use k=1? Perhaps the DC component is at the sweet spot which leads to a vanishing derivative and k=2 is used?

2. Can we add a clarification to Eq.(3) indicating the superscript k is the k-th harmonic in the definition of the external flux?

3. Does the phases of the AC flux drives need to be tracked, and how is that accomplished? The phase is important because it's being used as the signature for parity-dependence of the PST protocol. There have been several approaches to track the frames in two-qubit iswap gates (for example see arxiv 2308.08893). For state transfer perhaps the phases at the ends can be tracked in a similar fashion?

4. Does turning on the couplers simultaneously introduce unwanted next-nearest-neighbor interactions that could degrade

state transfer fidelity? Can this be detected and quantified in some way?

5. Can the authors comment on the scalability of their approach? From the transport data in Fig. 2 we see that even with decoherence effects included the simulation cannot fully capture the data, is the discrepancy due to unwanted ZZ or something else? What are some limiting factors in going to larger chains?

6. Another popular approach for perfect state transfer is to have identical couplings in the bulk of the chain and weak couplings at the ends (for example see PhysRevLett.106.040505), can the author comment on whether this is also a viable approach in their system?

Reviewer #4

(Remarks to the Author)
I co-reviewed this manuscript with one of the reviewers who provided the listed reports. This is part of the Nature Communications initiative to facilitate training in peer review and to provide appropriate recognition for Early Career Researchers who co-review manuscripts.

Version 1:

Reviewer comments:

Reviewer #1

(Remarks to the Author)
I thank the authors for their thoughtful response to my initial review. Overall, I continue to support the publication of this manuscript and I have no further questions for the authors.

Reviewer #2

(Remarks to the Author)
I appreciate the authors for addressing all the comments and making the necessary revisions. I am pleased that the authors' response has clarified the significance of the findings, which were initially unclear to me. Based on this, I would support the publication of this manuscript in Nature Communications.

Reviewer #3

(Remarks to the Author)
I am satisfied with the authors' response to my comments, I have no further objections.

Reviewer #4

(Remarks to the Author)
I co-reviewed this manuscript with one of the reviewers who provided the listed reports. This is part of the Nature Communications initiative to facilitate training in peer review and to provide appropriate recognition for Early Career Researchers who co-review manuscripts.

Open Access This Peer Review File is licensed under a Creative Commons Attribution 4.0 International License, which permits use, sharing, adaptation, distribution and reproduction in any medium or format, as long as you give appropriate credit to the original author(s) and the source, provide a link to the Creative Commons license, and indicate if changes were made.
In cases where reviewers are anonymous, credit should be given to 'Anonymous Referee' and the source.
The images or other third party material in this Peer Review File are included in the article's Creative Commons license, unless indicated otherwise in a credit line to the material. If material is not included in the article's Creative Commons license and your intended use is not permitted by statutory regulation or exceeds the permitted use, you will need to obtain permission directly from the copyright holder.
To view a copy of this license, visit https://creativecommons.org/licenses/by/4.0/

Below we provide a point-by-point response to the referees' comments (in blue).

Reviewer #1 (Remarks to the Author):

In this paper, the author provides an experimental demonstration of Perfect State Transfer (PST) in a 1D chain of qubits containing both zero and non-zero number of excitations. For the case where excitations are present in the system, the authors describe how the final transferred state will include a phase that depends on the parity of the total number of excitations in the chain. The authors then use the parity dependence of PST to generate a 3-qubits GHZ state with 88% fidelity (after accounting for single-qubit Z rotation).

This manuscript provides a nice experimental demonstration of Perfect State Transfer on a superconducting qubit platform, and discusses how one could apply this protocol in the presence of qubit excitations in the chain. The experimental data is of quality and supports the claims by the authors. Overall, I would support the publication of this manuscript in Nature Communications.

We thank the reviewer for their assessment.

Nevertheless I think the following questions could be interesting to address:

1. One advantage of performing this state transfer in the presence of qubit excitations is that it can be done while a state we wish to preserve is present on that chain (otherwise one could just reset the chain and do away with the parity dependence of the state transfer). The fact that one only needs to know about the parity of the qubits in the chain is interesting because in many error correction codes, the parities of the qubits is a known/measured quantity.

Indeed, the parity dependent properties of the state transfer suggest its possible utility in the context of quantum error correction. For example, we have begun investigations in the use of PST to efficiently map the stabilizers of data qubits to measure qubits. The naive approach would be to perform the same experiment as shown in Fig.3 of the manuscript where the parity of the data qubits is mapped to the phase of the transferred state. This would require a chain two qubits longer than the number of data qubits in the stabilizer. A better approach relies on the phase-kickback that the parity dependent transfer applies on the centre qubit in odd chains. This approach requires only one auxiliary qubit per stabilizer, making it more time- and qubit-efficient.

Two observations are important when considering the PST approach to stabilizer mapping:
1. Each PST operation swaps pairs of mirror-symmetric qubits. This rearrangement needs to be taken into account in the decoding of the error.
2. One always should compare to a two-qubit gate decomposition, which allows for efficient parallelisation of the measurement of all commuting stabilizers.
Indeed, two-qubit gates are more efficient for stabilizer measurements in the case of repetition codes. However, our ongoing investigation suggest that the PST operation can provide an advantage for the surface code. Using five qubit chains to map the four qubit stabilizers should result in a reduction by a factor of $5/\sqrt{32} \approx 0.88$ in the operation time. This can be further improved by using two sequential three qubit chains that can be parallelised over different stabilizers to obtain a reduction in time by a factor of $3/\sqrt{32} \approx 0.53$.

In this context,
a. Could this state transfer be done through a stabilized repetition code state without learning/disturbing the logical state?
b. What about a stabilized surface code? (some elements of this is addressed in Supplementary Note 5, but perhaps expanding on this protocol specifically in the presence of a stabilized surface code state would be interesting)
c. What is the effect of the state transfer on the qubits in the chain/lattice?

This is an interesting line of thought that we have not explored yet in detail. In general, the PST operation does not commute with the stabilizers of the repetition or surface codes because it applies the parity-dependent transfer on all mirror-symmetric sites. This effect can be accounted for either by appropriate modification of the decoding strategy or by the application of an additional smaller PST. Regarding the effect of PST on qubits in larger dimensions, we note that only the single-excitation dynamics produce a correct state transfer.

2. In Fig 2, it looks like the closer the qubit is to the center of the chain, the worse the state transfer fidelity is (as judged by the green bar plots in Fig 2 a,b,c). Presumably this has to do with the increased number of “paths” the information is travelling through (1 in a, 2 in b, and 3 in c). Is this expected, and if so how could it be mitigated?

We thank the referee for stimulating this investigation. While we had already performed extensive numerical analysis on the noise sensitivity of PST (below), we only studied the fidelity of the whole operation. We have since extended our study to find that, indeed, the transfer of the outer qubits is slightly more robust to frequency fluctuations than the transfers involving more “paths”.
Response Figure 1 shows the fidelity of the state transfer $|\langle\psi_j|U_{PST}|\psi_i\rangle|^2$ between mirror-symmetric sites as we introduce noise in the qubit frequencies (equivalent to detunings in the parametric drives). The outer qubit excitations transfer as a single wave-packet, making them seemingly more robust to frequency noise. Transfers from the intermediate and centre qubits are more sensitive, possibly due to the interference requirements of multiple paths during the dynamics.

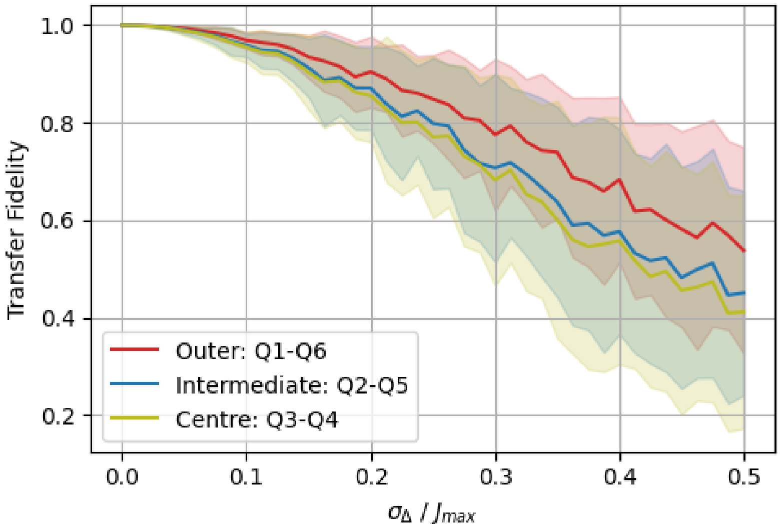

Response Figure 1. Transfer fidelity as a function of qubit frequency error. Qubit frequencies are sampled from a normal distribution of standard deviation $\boldsymbol{\sigma}_{\boldsymbol{\Delta}}$. Simulation parameters are set to match the 640ns transfer time of the PST in the manuscript. Shaded areas show standard deviation from 100 realizations of the sampled frequency errors.

Moreover, in our system, qubits 3 and 4 display a large hybridisation (due to their closeness in frequency) and measurement crosstalk, further deteriorating the precision of the PST protocol overall and specifically when initialising and measuring these two qubits.

3. In the paper, the use of PST for GHZ state preparation is compared with using “its decomposition into single- and two-qubit gates”. The authors find that PST is twice as fast. Besides this speed comparison, it could be interesting to know how the fidelity of the GHZ state from the two methods is expected to compare.

We thank the referee for raising this important point. Indeed, while errors due to relaxation and dephasing will scale trivially with time, the effect of coherent noise source is not obvious. Therefore, we have added Supplementary Note 4, which discusses the performance of PST and its optimal decomposition in the presence of ZZ errors and parameter fluctuations. In summary, we find that PST is more robust against parasitic ZZ couplings, as well as fluctuations in both coupling strengths and qubit frequencies. For further details, please refer to Supplementary Note 4.

Additionally, it’s worth noting that, if scalability and speed are the main motivating factors, there exist Measurement Based circuits for generating GHZ state which are of constant depth and could merit a mention (since for PST with a given maximum coupling strength, tau is inversely proportional to the chain length)

Mid-circuit measurements are indeed a powerful tool for generation of entangled states. We have added the following sentence in the discussion to highlight protocols using mid-circuit measurements “Besides purely unitary operations, we note that GHZ states can also be generated in constant depth via measurement-based circuits [79-84].”

Reviewer #2 (Remarks to the Author):

Key results

The authors present an experimental demonstration of Perfect State Transfer (PST) in a linear chain of six qubits with various configurations, including PST between all pairs of mirror-symmetric qubits. They also verify the parity dependence of PST with multiple excitations in the chain. Building on this parity dependence, the authors introduce a novel method for efficiently generating multi-qubit GHZ states in a system with only nearest-neighbor coupling. As a proof of concept, a three-qubit GHZ state is generated with a state fidelity of 88%.

Significances

This experiment showcases PST with some configurations that are not explored before, including the case where initial population at mid of the chain or transferring with multiple excitation within the chain. The application of PST for GHZ state generation could garner significant attention within the superconducting quantum computing community, as it offers a novel and efficient approach to non-local operations in superconducting circuits. However, it is important to note that PST is a well-established technique that has been extensively studied, both theoretically and experimentally. In fact, a similar, more challenging experiment has recently been published in Nature Communications, see the reference 51 in the main text. While the results here are interesting, they do not represent a significant advancement over prior work.

We thank the reviewer for their thoughtful assessment. Indeed, PST is a well-studied technique with multiple useful applications. However, experimental realisations of PST have been limited to the single-excitation regime. In this context, PST can be used to transfer entangled states, but not to generate them. In contrast, our work investigates PST's behaviour when applied to product states with arbitrary excitation numbers. We demonstrate the parity-dependent property that emerges in this scenario, which has not been previously observed. Additionally, using graph state theory, we showcase its potential for generating highly-entangled states.

From this perspective, we would like to specifically address the differences between our work and the reference mentioned by the referee, which also shows impressive results. Firstly, Xiang et al. (2024) implement the PST operation on a chain of qubits. However, this is only performed for transferring a single excitation between the outer qubits, as was shown in previous experimental realisations. The authors then continue by transferring three different states on a 2D grid (a single excitation state, a Bell state and a two excitation state). The state transfers need to be implemented separately since they require a different set of numerically optimised system parameters. We emphasise that these solutions are not the analytically-known PST operation. In particular, these operations do not function in the presence of other excitations in the system and do not generate entanglement. Therefore, we believe our work goes beyond the 1D and 2D realisations in Xiang et al. (2024) by explicitly demonstrating the entangling power of PST.

Presentation, Clarity and Validity

The manuscript is clearly written, and the experimental methods are presented in a comprehensible manner. The primary conclusions are well-supported by experimental data, which are collected and presented in a valid way. However, several key technical details are missing from the manuscript, as outlined in the comments to the authors below.

Comments to authors

1. While the manuscript successfully demonstrates parity-dependent PST across six qubits, the GHZ state generation is limited to three qubits. It would be helpful to include a discussion of the challenges in scaling this protocol to more qubits, as scalability is an important factor in evaluating the protocol's potential for practical use.

From the point of view of implementation, scaling PST to more qubits is straightforward. Increasing the number of qubits does increase the number of drive parameters that need to be simultaneously fine-tuned (two per parametric drive). However, previous experiments have shown simultaneous calibration of a large number of parameters (e.g. up to 55 parameters in [1]) so we are confident implementations with larger chains are possible.

On our device, GHZ states involving more qubits were expected to show significantly lower fidelities due to decoherence and ZZ errors. For example, simply taking into account errors during the PST operation the maximum fidelity expected for a 6 qubit GHZ is between 13% and 60%, depending on the choice of chain. Furthermore, due to the spurious ZZ interactions the single-qubit gates needed for state preparation and state tomography would also introduce significant errors, contributing to a large component of the measured errors. We, therefore, decided against performing these experiments and, instead, generated the GHZ and performed tomography on the three qubits with the lowest values of ZZ interaction, allowing us to reach close to the decoherence limit of F_sim=89.11%. We have added a clarifying statement to this regard in the manuscript.

To address the question of utility of the PST protocol we have compared its performance to that of its optimal decomposition into single- and two-qubit gates. Firstly, we note that the PST is at least twice as fast as its decomposition, making it less susceptible to relaxation and dephasing errors. Furthermore, we have studied the effect of coherent errors on the two implementations and found that PST is more robust against parasitic ZZ couplings, as well as fluctuations in both coupling strengths and qubit frequencies. These results have been added to the manuscript in Supplementary Note 4.

[1] M. Werninghaus, D. Egger, F. A. Roy, S. Machnes, F. Wilhelm and S. Filipp; Leakage reduction in fast superconducting qubit gates via optimal control. npj Quantum Information, 7(1), 14 (2021)

2. The authors attribute a significant portion of the error to residual ZZ interactions among qubits. However, a quantitative description of the strength of these residual ZZ interactions is lacking.

We thank the referee for highlighting this oversight. We have included the measured ZZ values in the device characteristics table.

3. Many of the results are presented in terms of qubit populations or quantum state tomography. However, the manuscript does not specify the readout fidelity, whether assignment errors were corrected before reconstructing the density matrix, or whether the density matrix reconstruction was done under physical constraints.

We thank the referee for highlighting these important points regarding measurement and the state tomography. Indeed, we utilise the techniques described by the referee. We have added the readout fidelities in the device characteristics table and added the following sentences in the manuscript: “Qubit populations are measured through dispersive readout. Measurement errors are characterised using an assignment matrix and mitigated by applying matrix inversion.”; “The density matrix is reconstructed

from the tomographic data using a maximum-likelihood estimator while imposing physical constraints.”.

4. Additionally, the quality of single-qubit operations should be discussed, as it is relevant for validating the quantum state tomography.

We have added the fidelities of single qubit operations in the device characteristics table.

5. Could the authors clarify the meaning of the vertical bars in Fig. 3c?

They refer to averaging over the four instances of the transfer experiment starting from different initial superpositions states (eigenvectors of X and Y operators). We have added a clarifying statement to the figure caption.

6. The effective Hamiltonian assumes the qubits as ideal two-level systems, but transmon qubits have only weak anharmonicity. At what point should higher energy levels be considered, and do they impose any limitations on the speed of PST operations?

The presence of higher levels is the source of spurious ZZ interactions which decrease the fidelity of the PST operation. However, the anharmonicity of the qubits is much larger than the coupling strengths achievable via parametric drive, therefore we don’t expect any limitation on speed due to leakage to higher excited states. Nonetheless, we note that these transition can still result in AC-Stark shifts modifying the strength of the ZZ interactions. These effects become relevant for high precision, when the static ZZ coupling is close to vanishingly small, and could be overcome by appropriate measurements [1] and pulsing techniques [1,2]. However, in our experiment the dynamic effect was much smaller that the static one.

[1] Y. Sung, L. Ding, J. Braumüller, A. Vepsäläinen, B. Kannan, M. Kjaergaard, ... and W. D. Oliver; Realization of high-fidelity CZ and ZZ-free iSWAP gates with a tunable coupler. Physical Review X, 11(2), 021058 (2021)

[2] M. Ganzhorn, D. J. Egger, S. Filipp, G. Von Salis and N. Moll; Cross-talk compensation in quantum processing devices, US Patent 10,452,991 (2019)

7. Figure 4a-d presents a novel illustration of how PST induces all-to-all connectivity and generates a GHZ state. However, this section is somewhat unclear. According to the figure, generating a six-qubit GHZ state requires two PST operations and an additional iSWAP operation between qubits 3 and 4. This seems to contradict a statement on line 288 of the main text, which claims "a single PST operation can be utilised to directly generate a GHZ state for any chain length."

We believe there is a misunderstanding: Fig. 4a shows how a single PST operation can be understood and decomposed as three parity-dependent iSWAP operations (for qubits 3 and 4 this operation reduces to a simple iSWAP). We have modified the figure to make this clearer.

8. According to Supplementary Table 1, the qubits used in the experiment have fixed frequencies, yet they exhibit substantial dephasing, with the shortest Ramsey decay time being only 6 μs. How does this dephasing affect the PST process?

Under Markovian noise, the dephasing acts a Lindblad operator, reducing the coherence of the multi-qubit state. However, for non-trivial noise spectra, the dephasing may affect the protocol differently. For example, we have performed simulations (Response to Referee #1 - Figure 1) showing that random qubit

detunings produce larger fidelity losses in transfers involving dynamics with multiple wave-packets. Nonetheless, as discussed above, we find that the PST process is more robust than its optimal decomposition to both coherent and incoherent sources of error.

9. The lowest qubit frequency in the experiment is 3.21 GHz, making it more susceptible to thermal noise. The manuscript would benefit from a discussion of how thermal noise may have impacted the experimental results.

Given our analysis techniques, we find that thermal noise does not significantly impact the outcome of the GHZ state fidelity. This is due to the use of measurement assignment error mitigation together with the physically constrained state estimation from tomographic data.

Furthermore, we note that the population distribution of superconducting qubits does not typically correlate with the temperature of the cryogenic environment for temperatures below a certain threshold [1, 2]. In our experiment, taking into account only the first two qubit levels, the temperatures as determined from on-off measurements varies from ~55mK to ~75mK.

While this inaccurate qubit ground state preparation does not affect the measured fidelity, it can be detrimental for more complex processes. Nonetheless, different techniques exist for qubit reset and qubit cooling that can be used to mitigate the problem.

[1] X. Y. Jin, A. Kamal, A. P. Sears, T. Gudmundsen, D. Hover, J. Miloshi, ... and W. D. Oliver; Thermal and residual excited-state population in a 3D transmon qubit. Physical Review Letters 114, 24:240501, (2015)

[2] D. S. Lvov, S. A. Lemziakov, E. Ankerhold, J. T. Peltonen, J. P. Pekola; Thermometry Based on a Superconducting Qubit. ArXiv: 2409.02784 (2024)

Reviewer #3 (Remarks to the Author):

This is a nice paper demonstrating perfect state transfer in a superconducting system utilizing fixed-frequency transmons with tunable couplers. A highlight of the work is the entanglement generation via parity-dependent state transport. While the authors only generated a three-qubit GHZ state, I like the experimental protocol because it provides a novel alternative to quantum circuits using two-qubit gates. The paper is well written and the data are convincing, however I would like to see more clarifications on the following points.

We thank the reviewer for their assessment.

1. The qubit-qubit coupling are engineered by pulsing on the tunable coupler with AC and DC components described in line 93. How is the DC component chosen? The AC component is modulated at the k-th harmonic, what k is used experimentally for each coupling? Supp. Fig. 1 shows a 650/750 MHz filter on the flux line which is more than enough to drive the k=1 harmonics for all pairs of qubits, is there any reason to not use k=1? Perhaps the DC component is at the sweet spot which leads to a vanishing derivative and k=2 is used?

The couplers were all biased in the upper sweet spot for the measurements shown in the main manuscript. The k=2 harmonic was used for the parametric drives for exactly the reason given by the reviewer. We have found that while this biasing is not always optimal for the spurious ZZ interaction, it was necessary for reducing the effect of decoherence introduced by the flux-sensitive coupler both when idling and especially when driving the parametric interactions.

2. Can we add a clarification to Eq.(3) indicating the superscript k is the k-th harmonic in the definition of the external flux?

We thank the referee for highlighting this oversight. We have updated the text accordingly.

3. Does the phases of the AC flux drives need to be tracked, and how is that accomplished? The phase is important because it's being used as the signature for parity-dependence of the PST protocol. There have been several approaches to track the frames in two-qubit iswap gates (for example see arxiv 2308.08893). For state transfer perhaps the phases at the ends can be tracked in a similar fashion?

Indeed, the tracking of the difference frame is important for the iSWAP gate and the PST protocol. In our experiment the phases are tracked by synchronising the MW drives of the qubits and the flux drives of the couplers. However, we note that while this phase relation is indeed important for the final state, the parity-induced phase is independent of this offset phase, and could be harnessed for example in an interferometry experiment.

4. Does turning on the couplers simultaneously introduce unwanted next-nearest-neighbor interactions that could degrade state transfer fidelity? Can this be detected and quantified in some way?

Next-nearest-neighbour hopping terms would appear if the drives satisfy specific conditions akin to Raman drives, but these are highly unlikely. We note that, in general, small higher-order longitudinal couplings (Z...Z) will always appear, even in the static case [1].
Nevertheless, simultaneous neighbouring flux drives yield a shift to the coupler and qubits that must be accounted for by a further calibration, as described and demonstrated in the method section of the

manuscript. Furthermore, as in previous experiments [2], we observe minimal AC flux crosstalk, which could otherwise cause non-trivial errors.

[1] X. Xu, C. Vignes, M.H. Ansari and J. Martinis, Lattice Hamiltonians and Stray Interactions Within Quantum Processors. arXiv:2402.09145 (2024)
[2] D. M. Abrams, N. Didier, S. A. Caldwell, B. R. Johnson and C. A. Ryan; Methods for measuring magnetic flux crosstalk between tunable transmons. Physical Review Applied, 12(6), 064022 (2019)

5. Can the authors comment on the scalability of their approach? From the transport data in Fig. 2 we see that even with decoherence effects included the simulation cannot fully capture the data, is the discrepancy due to unwanted ZZ or something else? What are some limiting factors in going to larger chains?

For Figure 2 we believe that the discrepancy is due to dephasing and 1/f noise on the Hamiltonian parameters, which were not included in the simulation.
In general when considering multiple excitation (as is the case for the experiments in Figure 3 and Figure 4 of manuscript) unwanted ZZ errors can limit the fidelity of the PST operation. While ZZ errors played a role in our experiments, these can be suppressed by proper targeting of qubit and coupler parameters.

To assess the scalability of our approach, we have compared its performance and that of its optimal decomposition in the presence of a variety of noise sources. The numerical simulations show that PST is more robust to fluctuations in couplings and qubit frequencies, as well as to the presence of unwanted ZZ interactions. Furthermore, the lower operation time of the PST protocol makes it less sensitive to relaxation and dephasing errors. These results are discussed in detail in Supplementary Note 4.

6. Another popular approach for perfect state transfer is to have identical couplings in the bulk of the chain and weak couplings at the ends (for example see PhysRevLett.106.040505), can the author comment on whether this is also a viable approach in their system?

This approach is indeed interesting and is definitely viable in our system. However, the reduction in control complexity results in an increase in operation time. Therefore, the suggested approach would be more advantageous in tailor-made systems with large static uniform couplings in the bulk of the chain.

Reviewer #4 (Remarks to the Author):

I co-reviewed this manuscript with one of the reviewers who provided the listed reports. This is part of the Nature Communications initiative to facilitate training in peer review and to provide appropriate recognition for Early Career Researchers who co-review manuscripts.

We thank the referee for their contribution.
